# Base Excision Repair in the Immune System: Small DNA Lesions With Big Consequences

**DOI:** 10.3389/fimmu.2020.01084

**Published:** 2020-05-29

**Authors:** Maria Stratigopoulou, Tijmen P. van Dam, Jeroen E. J. Guikema

**Affiliations:** Department of Pathology, Lymphoma and Myeloma Center Amsterdam (LYMMCARE), Amsterdam UMC, University of Amsterdam, Amsterdam, Netherlands

**Keywords:** base excision repair (BER), germinal center (GC), lymphoma, autoimmune diseases, class switch recombination (CSR), somatic hypermutation (SHM)

## Abstract

The integrity of the genome is under constant threat of environmental and endogenous agents that cause DNA damage. Endogenous damage is particularly pervasive, occurring at an estimated rate of 10,000–30,000 per cell/per day, and mostly involves chemical DNA base lesions caused by oxidation, depurination, alkylation, and deamination. The base excision repair (BER) pathway is primary responsible for removing and repairing these small base lesions that would otherwise lead to mutations or DNA breaks during replication. Next to preventing DNA mutations and damage, the BER pathway is also involved in mutagenic processes in B cells during immunoglobulin (Ig) class switch recombination (CSR) and somatic hypermutation (SHM), which are instigated by uracil (U) lesions derived from activation-induced cytidine deaminase (AID) activity. BER is required for the processing of AID-induced lesions into DNA double strand breaks (DSB) that are required for CSR, and is of pivotal importance for determining the mutagenic outcome of uracil lesions during SHM. Although uracils are generally efficiently repaired by error-free BER, this process is surprisingly error-prone at the *Ig* loci in proliferating B cells. Breakdown of this high-fidelity process outside of the *Ig* loci has been linked to mutations observed in B-cell tumors and DNA breaks and chromosomal translocations in activated B cells. Next to its role in preventing cancer, BER has also been implicated in immune tolerance. Several defects in BER components have been associated with autoimmune diseases, and animal models have shown that BER defects can cause autoimmunity in a B-cell intrinsic and extrinsic fashion. In this review we discuss the contribution of BER to genomic integrity in the context of immune receptor diversification, cancer and autoimmune diseases.

## Introduction

The adaptive immune response is of crucial importance for the elimination of pathogens, such as bacteria, viruses, and other foreign substances. Lymphocytes are the prime mediators of the adaptive immune response, recognizing antigens by their specific antigen receptors (AgR). DNA recombination and mutation processes ensure the generation of a vast array of AgRs. During lymphocyte development, the antigen-independent recombination of variable (V), diversity (D), and joining (J) gene segments assembles the genetic code for the T-cell receptor (TCR) and the B-cell receptor (BCR). This highly ordered process involves the generation of DNA double-strand breaks (DSB) followed by non-homologous end-joining (NHEJ) of the various gene segments. This process is unique for lymphocytes and requires the DNA nicking activity of recombination activating gene products 1 and 2 (RAG1, RAG2) (1).

B cells can undergo additional diversification processes that shape the immunoglobulin (Ig) repertoire in an antigen-dependent fashion. Class switch recombination (CSR) is an (predominantly) intrachromosomal looping process by which the constant region coding for the Ig isotype is exchanged, thereby altering the effector function of the expressed Ig. Switch regions that are located upstream of each *Ig* constant region are the targets for DSBs that are resolved by NHEJ, resulting in the looping out of DNA intervening the switch regions from upstream and downstream constant regions (2). Somatic hypermutation (SHM) is a crucial event for antibody affinity maturation. Point mutations are introduced in the recombined V(D)J and *Ig* switch regions. B cells with improved affinity for antigen as a result of these mutations are clonally selected to differentiate into memory B cells and plasma cells by competing for antibody-mediated antigen capture and subsequent acquisition of T-cell help within germinal centers (GC) in secondary lymphoid organs (3). CSR and SHM are initiated by the activation-induced cytidine deaminase (AID) (4, 5). AID instigates both events by provoking base damage directed at cytosines (C), generating deoxy-uracil (U) that triggers mutagenic processing by the base excision repair (BER) and mismatch repair (MMR) pathways, resulting in point mutations and DSBs.

**Figure 1:**
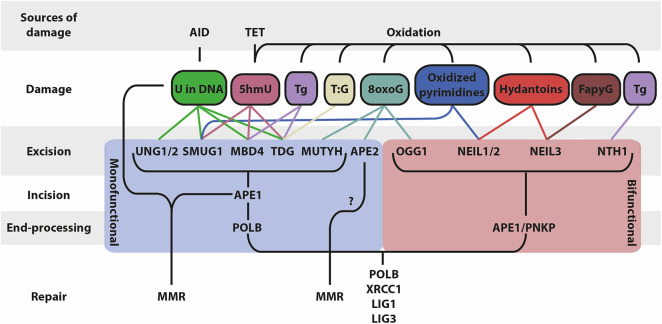
Schematic overview of BER and associated factors. BER functions on different types of DNA base lesions that are generated by AID, TET and through oxidation. BER occurs in four mains steps that differ based on the mono/bifunctionality of the glycosylase: (i) base excision, (ii) DNA backbone incision, (iii) DNA end processing, (iv) repair of the lesion (5hmU, 5-hydroxymethyluracil; Tg, thymine glycol; 5hmC, 5-hydroxymethylcytosine; 8oxoG, 8-oxoguanine; FapyG, 2,6-diamino-4-hydroxy-5-formamidopyrimidine).

**Figure 2:**
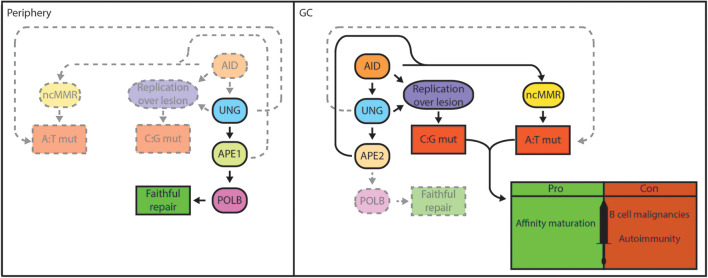
BER functions as a double-edged sword in B cells. Genomic integrity is safeguarded by the BER pathway in lymphocytes in the periphery. Damaged bases are faithfully repaired by UNG, APE1, and POLB in case of short-patch BER **(left)**. In GC B cells, localized base damage is introduced in the *Ig* genes by AID **(right)**. Some localized uracil lesions generated by AID overwhelm/escape BER and are replicated resulting in C:G transitions, but may also trigger A:T mutations. Others are engaged by UNG and are converted into AP sites that can escape processing by BER and are replicated by REV1, resulting in C:G transversions. Further processing of AP sites by BER generates nicks that serve to promote patch excision and mutagenic repair by ncMMR, which is responsible for the majority of A:T mutations. Mutagenic repair by ncMMR in GC B cells is stimulated by low POLB protein expression. AID-instigated mutations contribute to affinity maturation and immune function, but can also result in genomic instability that underlie B-cell malignancies or generate autoantibodies that cause autoimmune diseases.

Typically, BER is initiated by the recognition and removal of damaged bases by DNA glycosylases resulting in the formation of apurinic/apyrimidinic (AP) sites. These AP sites are highly mutagenic and require subsequent processing by AP endonucleases or by the AP lyase activity of bifunctional glycosylases, which nick the phosphodiester backbone of the AP site. The resulting DNA single-strand nicks can be processed into DSBs or be repaired by displacement synthesis (long-patch BER) or non-displacement synthesis (short-patch BER) (6, 7) (Figure 1). Interestingly, MMR is a primarily replication-linked repair pathway that acts on the same base lesions as BER. The three important steps that constitute the MMR pathway are: (i) mismatch recognition by MutS homolog (MSH) heterodimers (typically MSH2/MSH6; MutSα); (ii) recruitment of MutL homolog 1 (MLH1) and post-meiotic segregation-increased homolog 2 (PMS2) heterodimers (MutLα) and exonuclease 1 (EXO1), which are involved in the excision of a patch containing the damaged base(s); (iii) recruitment of DNA polymerases and fill-in synthesis (8). However, MMR can also act independently of DNA replication (9, 10). Importantly, in B cells undergoing CSR, AID-generated U:G mismatches give rise to MMR-dependent DSBs in the G1 phase of the cell cycle by patch excision of the mismatch-containing strand until a DNA nick on the opposite strand is reached (9). In addition, in B cells undergoing SHM, MMR displays a non-canonical (mutagenic) activity by the specific recruitment of the error-prone translesion polymerase POLH, which lacks proofreading activity. The error-prone activity of POLH is responsible for mutations at adenosine (A) and thymidine (T) bases during SHM, complementing a full spectrum of DNA mutations triggered by AID (11–13). The mechanistic basis for the switch to mutagenic non-canonical MMR (ncMMR) in B cells remains to be fully elucidated, and whether it is restricted to the G1 phase is currently unknown. However, *in vitro* and *in vivo* experiments indicate that the monoubiquitination of proliferating cell nuclear antigen (PCNA) is linked to ncMMR activity and is of crucial importance for mutations at A:T bases during SHM (10, 14, 15). Apparently, AID-dependent base lesions evade faithful DNA repair and elicit mutagenic repair, which critically involves BER and MMR (Figure 2).

Mutagenic repair can generate genomic alterations such as chromosomal translocations, and lead to the activation of oncogenes or inactivation of tumor suppressors. AgR diversification mechanisms are associated with hallmark genetic aberrations in B-cell lymphomas, mostly derived from illegitimate CSR events and off-target mutations caused by AID (16, 17). Mutagenic repair is coupled to a partial inactivation of the short-patch BER pathway in rapidly dividing GC B cells (Figure 2), which contributes to genomic instability (18) (preprint). Accordingly, the majority of human B-cell lymphomas are derived from the GC (19).

Autoimmune diseases are characterized by a breach of clonal tolerance that regulates the activation and survival of B cells independent of antigen specificity, often resulting in autoantibody production, which plays a pivotal role in the pathogenesis of these diseases (20). Isotype-switched autoantibodies are of crucial importance in several autoimmune disease models. Loss of AID function was shown to ameliorate disease manifestations in mouse models for multiple sclerosis (MS) (21, 22) and systemic lupus erythematosus (SLE) (23, 24), although from these studies it can not be concluded whether this is related to loss of CSR or SHM, as both require AID. However, in a recent study the effects of CSR versus SHM on the development of MS in a mouse model were assessed using an elegant approach, showing that CSR was of major importance for disease incidence and severity (25). Contrastingly, SHM activity was shown to be involved in the clonal redemption of anergic autoantibody-expressing B cells in humans and in autoantibody-dependent disease models in the mouse, by causing mutations that steer away from self-reactivity (26–28). Moreover, DNA repair pathways, including BER, have been linked to autoimmunity by suppressing the release of DNA, which is involved in the pathogenesis of autoimmune diseases (29, 30). DNA repair deficiencies may lead to the increased formation of endogenous DNA damage, which provokes apoptosis and the release of DNA fragments that can stimulate innate inflammatory responses. Upon endocytosis of extracellular DNA, engagement of endosomal and intracellular DNA sensors such as Toll-like receptor 9 (TLR9) and the cyclic-di-GMP-AMP synthetase (cGAS) results in the activation of the stimulator of interferon genes (STING) adaptor protein, which stimulates the release of proinflammatory cytokines that may contribute the pathogenesis of autoimmune diseases (31).

In this review we discuss the involvement of BER in lymphocyte development and function, with a particular focus on diseases such as lymphomas, leukemias, and autoimmune disorders.

## DNA Glycosylases

DNA bases damaged by spontaneous and enzymatic deamination, depurination, oxidation or alkylation are repaired by the BER pathway. As an initial step, the damaged base is recognized and removed from the DNA backbone by one of the DNA glycosylases. Monofunctional glycosylases remove the damaged base and leave an AP site, which requires subsequent cleaving of the DNA backbone by an AP endonuclease, whereas bifunctional glycosylases possess both glycosylase and AP lyase activity. The various glycosylases recognize different types of damaged bases (Figure 1). Deamination of C to U is the most common base damage, which can be repaired by the monofunctional glycosylases UNG, TDG, MBD4, or SMUG1 (32). These enzymes have overlapping and distinct functions, mostly related to different substrate preferences (Table 1).

**Table 1:** An overview of biological and chemical attributes of BER proteins.

	**Enzyme**	**Substrates**	**Function**	**Developmental defect in mice**	**Immune defect**	**Disease in mice**	**Disease in humans**	**Mouse KO references**
Glycosylases
	UNG2	U in DNA	Monofunctional	Viable; increased levels of U in dividing cells	Abrogated CSR	*Ung2^−/−^* 20-fold increase in lymphoid hyperplasia and B-cell lymphomas; deficiency increased hyper-IgM syndrome; *Ung2^−/−^ Msh2^−/−^* accumulation of AID-dependent mutations in non-*Ig* target genes.	UNG deficiency causes hyper-IgM syndrome	(33)
	UNG1	U in DNA	Monofunctional	–	–	–	–	–
	SMUG1	5FoU, 5CaU, U; 5hmU in DNA and ssRNA;	Monofunctional	Viable	Co-deletion in Ung2^−/−^ mice further decreases CSR	No causative link between lymphoma or autoimmunity.	mRNA levels predicted therapy response in breast cancer, gastric cancers and colorectal cancer; SNPs associated with increased bladder cancer risk	(34)
	TDG	T/U:G; Tg in Tg:G	Monofunctional	Lethal	–	No causative link between lymphoma or autoimmunity.	–	(35)
	MBD4	Tg/T:G; U, 5hmU	Monofunctional	Viable; depletion increased C>T at CpG	Severely reduced CSR; Increased SHM in DT40 cells	Increased frequency of intenstinal tumors in APC+/min mice	Polymorphisms associated with cancer; loss of *MBD4* in AML increased mutational burden >30-fold	(36)
	OGG1	8-oxoG	Bifunctional	Viable and fertile; accumulation of 8-oxoG; increased spontaneous mutations	–	Accumulation of 8-oxoG; increased lung cancer in mice; *Ogg1^−/−^ Mutyh^−/−^* mice further predisposed to cancer	Polymorphisms in human associated with cancer and autoimmunity	(37)
	MUTYH	A in A:8-oxoG	Monofunctional	Viable and fertile; susceptible to oxidative stress	–	KO predisposed to cancer; *Ogg1^−/−^ Mutyh^−/−^* mice further predisposes to cancer	Human variants of *MUTYH* are associated with RA and predisposed to CRC	(38)
	NTH1	Tg	Bifunctional	Viable and fertile; slower Tg turnover in liver	–		A human variant of *NTH1* is related to genomic instability	(39)
	NEIL1	Hydantoins; oxidized pyrimidines	Bifunctional	Viable and fertile; develop severe metabolic syndrome by 6–10 months	Decreased GC B-cell expansion; decreased Ag-specific Ab titers	Combined deficiencies of *Neil1/2/3* did not predispose to cancer	Protein variants correlated to cancer; no direct causative link	(40)
	NEIL2	Similar to NEIL1	Bifunctional	Viable	–	–	–	(41)
	NEIL3	Hydantoins; FapyG in ssDNA	Bifunctional	Viable and fertile	Increased GC B-cell apoptosis	Combined deficiency of *Neil1/2/3* did not predispose to cancer in mice; *Neil3* deficiency in mice increases autoimmunity	Protein variants correlated to cancer; no direct causative link	(42)
AP endonucleases
	APE1	AP sites; oxidized C	Endonuclease; transcriptional regulator (CSR)	Lethal	Heterozygous deletion increased mutations and reduced CSR	–	Human polymorphisms linked to cancer; Possible link to SLE	(43)
	APE2	AP sites; A in A:8-oxoG	3′-5′ exonuclease; 3′-phosphodiesterase; endonuclease (CSR, SHM, HR, NHEJ)	Viable	*Ape2Y/-* 2-fold decrease of pre-B cells and mature B cells, smaller GCs; SHM: Reduced A:T mutations; B cells hypersensitive to oxidative damage	–	APE2 variants in multiple human cancers; mRNa level associated with DDR status	(44)
Nick processing enzymes
	POLB	Abasic sites with 3′OH and 5′dRP	DNA synthesis; dRP lyase activity	Lethal; hypomorphic mice increased SHM and GC amount	–	Aging heterozgyous mice increased lymphoid hyperplasia and lymphoma incidence.	POLB variants implicated in cancer and autoimmunity	(45)
	FEN1		LP-BER, DNA replication; MMEJ, HR	Lethal	*Fen1^+/−^* dimished lymphocyte cellularity	*Fen1^+/−^* develop B-cell lymphomas (17%); increased intestinal adenocarcinomas in *Apc-mut* mice; LOF mutant predisposed to autoimmunity	Overexpression and LOF mutations are associated with multiple cancers	(46)
	LIG1		LP-BER, DNA replication; MMEJ	Lethal	SHM reduced	–	*LIG1* deficiency causes lymphopenia; spectrum of immune deficiencies	(47)
	LIG3		SP-BER, MMEJ; mitochondrial DNA maintenance	Lethal	–	–	–	(48)

*LOF, loss-of-function; CRC, colorectal cancer; RA, rheumatoid arthritis; DDR, DNA damage response*.

### Uracil DNA Glycosylase (UNG)

The *UNG* gene encodes two isoforms that differ by their N-terminal sequence and localize to the mitochondria (UNG1) and the nucleus (UNG2) (49). Due to its high turnover rate UNG2 is the predominant glycosylase in U removal and is active on both single-stranded (ss) and double-stranded (ds) DNA, while TDG and MBD4 act on dsDNA (50–52), and SMUG1 preferentially acts on ssDNA (53). The expression of UNG peaks in S-phase, but there is sufficient evidence that UNG also acts at the G1-S transition of the cell cycle, which is especially relevant for U removal during CSR and SHM in B cells (9, 54).

UNG2 is the major DNA glycosylase involved in CSR and SHM, while SMUG1 has a backup function (55, 56). The homozygous deletion of UNG is not inherently lethal to embryonic development in mice. *Ung*^−/−^ mice display a slow removal of U and increased steady-state levels of genomic U in dividing cells, whereas only a slight increase in the spontaneous mutation frequency was observed (57). Lymphocyte development appears to be unperturbed in *Ung*^−/^^−^ mice, although a fraction (~20%) of aging *Ung*^−/^^−^ mice develop lymphoid hyperplasia, which generally precedes the development of lymphomas in these animals. *Ung*^−/^^−^ mice display an ~20-fold increased risk of developing B-cell lymphomas (58, 59). Importantly, *ex vivo* CSR to IgG3 and IgG1, induced by lipopolysaccharide (LPS) or LPS and interleukin-4 (IL4) is nearly abrogated (~10% of wildtype levels) in the absence of UNG (33). Interestingly, in mice UNG deficiency had minimal impact on the basal serum levels of Ig subclasses *in vivo*, whereas neutralizing switched Ig levels were severely (~100-fold) diminished in response to acute vesicular stomatitis virus (VSV) infection in mice. These results indicate that during chronic antigen exposure switched Ig originating from infrequent UNG-independent CSR can accumulate, whereas acute antigen exposure requires UNG for the timely and efficient generation of neutralizing switched Ig (60).

It was shown that UNG is required for the generation of DSBs in Ig S regions by processing AID-generated Us into nicks, which when in close vicinity on either DNA strand result in DSBs, or when further apart, require the MMR pathway to be converted into DSBs (61–63). During SHM, UNG plays a key role in the generation of C/G transversions, whereas C to T transition mutations are increased in *Ung*^−/^^−^ mice (64). Ensuing work has shown that REV1 acts downstream of UNG to cause C/G transversions, bypassing AP sites by its translesion cytidyl transferase activity (65). In addition, UNG counteracts the accumulation of AID-generated Us that instruct the insertion of A on the other strand, resulting in C to T and G to A transitions. Mutations at A and T bases are also diminished in *Ung*^−/^^−^ mice (33). The vast majority of A:T mutations during SHM depend on non-canonical MMR (ncMMR), where the U:G mismatch is recognized by MSH2/MSH6 heterodimers that recruits EXO1 to excise a patch of DNA that contains the U (66). However, EXO1 activity requires a pre-existing nick, which is provided by UNG and AP endonuclease activity. Of interest, the PMS2/MLH1 heterodimer also possesses endonuclease activity that nicks 5′ from U:G mismatches (67, 68). Perhaps this MMR-dependent nicking activity may partly explain the relatively unperturbed basal Ig levels in UNG deficient mice, by allowing infrequent UNG-independent CSR that accumulates over time due to chronic antigen exposure (60). PMS2 deficiency in mice had a negligible effect on A:T mutagenesis (68), but PMS2 may act as a backup in UNG deficient B cells, as A:T mutations were ~50% reduced in *Ung*^−/^^−^ *Pms2*^−/^^−^ mice (69). Despite the apparent role of UNG in the generation of the full spectrum of AID-induced mutations during SHM, antibody affinity maturation toward a complex antigen such as keyhole limpet hemocyanin (KLH) seems to be intact in UNG deficient animals (60). The mechanistic involvement of UNG in SHM and CSR has been largely corroborated in human subjects that lack UNG due to gene mutations (70–72). However, a hyper-IgM phenotype is frequently observed in human UNG deficient patients, whereas this was not apparent in *Ung*^−/^^−^ mice. This may reflect a species-specific difference or perhaps a clinical bias caused by the mere fact that patients with severe immunodeficiency are more Likely to be identified due to clinical symptoms, while patients with mild symptoms remain largely undetected.

The role of UNG in lymphomagenesis has been addressed in several studies. Evidence for the promiscuous targeting of AID responsible for mutations in non-*Ig* loci was provided in *Ung*^−/^^−^ *Msh2*^−/^^−^ deficient mice (73). Due to the lack of repair or mutagenic processing, the accumulation of AID footprint mutations (74) was observed in various non-*Ig* genes, including B-cell lymphoma-associated oncogenes such as *Bcl6, Pim1*, and *c-Myc* (73). It was postulated that BER and MMR both protect the genome from AID off-target activity that may contribute to lymphomagenesis (75), but more recent data suggest that MMR has a dominant function in that regard (76). In addition, UNG was shown to be involved in the repair of AID-induced DNA damage at telomeres (77). In accordance, about 20% of aging *Ung*^−/^^−^ mice develop lymphoid hyperplasia that may progress to B-cell lymphomas in about half of these mice when aged beyond 18 months. Based on histological features these lymphomas were classified as high-grade follicular lymphoma (FL) (58). Lymphoid hyperplasia was observed in some of the human subjects lacking UNG (2 out of 3), however, the rarity of this condition precludes any firm conclusions on whether UNG deficiency causes lymphomas in humans (71, 78). In contrast, UNG deficiency was shown to be protective for the development of BCL6-driven mouse diffuse large B-cell lymphomas (DLBCL), whereas MSH2 deficiency or the combined deficiency of UNG and MSH2 accelerated lymphomagenesis, accompanied by the accumulation of AID-dependent mutations in non-*Ig* target genes (76). These data suggest that MSH2 has a strong role in preventing mutations, whereas UNG is actually involved in the generation of mutations (downstream of AID) in BCL6-driven mouse lymphomas. In a c-Myc-driven lymphoma mouse model the deficiency of UNG had no impact on lymphomagenesis (79). These results underscore the dual character of BER in AID-instigated lesions, contributing to both faithful repair and mutagenic processing, depending on the context. How the balance between these contrasting outcomes is regulated is an important outstanding question in the field.

The involvement of UNG in autoimmune diseases is complex, as paradoxically, immunodeficiency (such as associated with the loss of UNG) is linked to autoimmune disease by multiple (indirect) means, for example by the loss of peripheral tolerance. Several excellent recent reviews discuss the potential mechanisms of immunodeficiency-related autoimmune diseases, which goes beyond the scope of this review (80–83). In a recent experimental model the connection between UNG and autoimmune disease was illustrated, showing that the loss of UNG resulted in a lower disease severity in the experimental autoimmune encephalomyelitis (EAE) mouse model for MS, which most likely is attributable to the reduction in isotype-switched autoantibodies (25). Interestingly, UNG deficiency in humans had no apparent effect on the frequency of autoreactive naïve B cells, whereas these were increased in AID-deficient subjects. These results indicate that AID is involved in a peripheral B-cell tolerance checkpoint that is related to SHM, but not CSR, as UNG deficient B cells can still undergo SHM but have severely impaired CSR (84).

### Single-Strand Selective Monofunctional Uracil Glycosylase (SMUG1)

SMUG1 is monofunctional glycosylase that is involved in the removal of pyrimidine oxidation products such as 5-formyluracil (5FoU) and 5-carboxyuracil (5CaU) (85, 86), and is also involved in the removal of 5-hydroxymethyluracil (5hmU) from DNA, which is an oxidation product of thymine (34, 87). The ten eleven translocation (TET) dioxygenase enzymes can catalyze the conversion from T to 5hmU (88, 89) (Table 1; Figure 1). This lesion can also be present in ssRNA and be removed by SMUG1 (90). SMUG1 associates with a ribonucleoprotein complex in nucleoli and Cajal bodies and is involved in RNA quality control and co-transcriptional processing by removing 5hmU from ribosomal RNA and telomeric RNA, which is required for proper telomerase activity (90, 91). In addition, SMUG1 can be involved in the repair of U:G mismatches, and is the main means of U excision in *Ung*^−/−^ mice (92). Strikingly, the expression of SMUG1 is much higher in mouse than in human cells and is not cell cycle-regulated (93). SMUG1 appears not to be directly involved in AgR diversifications. However, SMUG1 is capable of performing a backup function in the absence of UNG. For instance, the residual CSR in *Ung*^−/−^ mice is significantly decreased by concomitant loss of SMUG1. Serum IgG3, IgG2b, and IgA levels were decreased ~4-, 10-, and 2-fold in *Ung*^−/−^ *Smug1*^−/−^ mice compared to *Ung*^−/−^ mice at the age of 6 months, whereas IgG1 levels appeared to be unaffected. However, in 6 weeks-old *Ung*^−/−^ *Smug1*^−/−^ mice IgG1 levels showed an ~3-fold decrease compared to *Ung*^−/−^ mice. *In vitro* IgG1 CSR was reduced 2–3-fold in *Ung*^−/−^ *Smug1*^−/−^ compared to *Ung*^−/−^ splenic B-cell cultures, suggesting that the kinetics of SMUG1-dependent CSR is considerably slower *in vivo* (51). Moreover, *ex vivo* IgG1 CSR in *Ung*^−/−^ B cells was restored by retroviral SMUG1 overexpression to a similar level as by retroviral UNG overexpression (22 vs. 17% IgG1 CSR, respectively) (94). Combined deficiencies for UNG and SMUG1 further reduced A:T mutagenesis (54% A:T mutations in the JH4 intron in wildtype mice; 55% in *Smug1*^−/−^, 46% in *Ung*^−/−^, 39% in *Ung*^−/−^ *Smug1*^−/−^), indicating that SMUG1 can partially compensate for the loss of UNG by excision of U in the vicinity of U:G mismatches resulting in nicks required for patch excision (56). In agreement, SMUG1 overexpression in mice double deficient for MSH2 and UNG affected the SHM pattern, partly restoring the loss of C/G transversions and A:T mutations in *Msh2*^−/−^ *Ung*^−/−^ mice to about 50% of wildtype levels. *Ex vivo* IgG1 CSR is nearly ablated in *Msh2*^−/−^ *Ung*^−/−^ splenic B cells, but was restored to about 15–20% of wildtype levels when SMUG1 was overexpressed in these cells (55). It was shown that the endonuclease activity of PMS2 is responsible for the residual A:T mutagenesis in UNG and SMUG1 deficient B cells, but whether loss of PMS2 further diminishes A:T mutations in *Ung*^−/−^ *Smug1*^−/−^ mice remains to be tested (69). In conclusion, under normal conditions SMUG1 does not participate in CSR or SHM and is solely involved in these processes in cells that lack UNG activity.

As of yet, there are no studies available that implicate SMUG1 in lymphomagenesis or autoimmune diseases. However, the expression and the genetic variation of *SMUG1* have been studied for different type of human cancers. *SMUG1* mRNA expression negatively correlated with aggressive disease and survival in breast cancer, and predicted response to adjuvant therapy (95). Human gastric cancers with microsatellite instability are characterized by the downregulation of *SMUG1* transcription, which could be related to the response to therapy (96). In addition, an association between bladder cancer and a SNP in the *SMUG1* gene was reported (97). In mice deficient for SMUG1 and UNG there is accumulation of genomic U. Whole genome sequencing of UNG/SMUG1 deficient tumors revealed increased mutations, consisting primarily of C to T transitions within CpG sequences, which can be considered as a mutational signature for those tumors (98).

### Thymine DNA Glycosylase (TDG)

TDG can repair T:G mismatches that arise from 5-methylcytosine (5mC) deamination (99). However, it has more pronounced activity toward U:G mismatch containing substrates *in vitro* (Table 1; Figure 1) (50, 51). However, whether TDG can excise U from U:G mismatches *in vivo* remains to be established. TDG is expressed at low levels in S-phase as it remains bound very tightly to the AP site after excision of U or T from the mismatch, which may stall the replication fork (100). In addition, TDG performs an important function in the transcriptional regulation of developmental genes by interacting with transcriptional cofactors at the promoters of these genes, and is essential for embryonic development (35). It was demonstrated that TDG is crucial for active demethylation and protection of CpG islands from hypermethylation. TDG associates with AID, and it was proposed that AID-dependent deamination of 5mC and 5hmC generates the substrates for TDG-mediated base removal and repair, thereby erasing the DNA methyl marks (101).

Despite its presumed association with AID, the exact role of TDG in AgR diversification mechanisms in B cells has not been studied extensively. It was shown that retroviral overexpression of TDG was not able to restore *ex vivo* CSR in UNG deficient B cells (94). TDG can only remove U from DNA when mispaired with G in dsDNA. We speculate that TDG perhaps is not be involved in CSR due to the fact that U excision from dsDNA is not efficient in triggering CSR. However, a possible role of TDG in AgR diversification *in vivo* remains unexplored. This would require a B-cell-specific conditional knockout model, which has not been reported (yet). Nonetheless, a mouse strain carrying a floxed *Tdg* allele is available, showing that the conditional knockout of *Tdg* in intestinal epithelial cells resulted in a 2-fold increase in adenomas in the small intestine of female tumor-predisposed *APC*^*min*/+^ mice (102). This effect was attributed to the fact that TDG is a transcriptional coregulator of the estrogen receptor (103, 104), which has a protective effect on intestinal tumor formation (105).

It is conceivable that TDG is involved in aberrant DNA methylation in various cancers including lymphomas and leukemias, but conclusive data to support this is lacking. There are some indications that TDG may have a role in the malignant plasma cell neoplasia multiple myeloma (MM). In MM cell lines it was observed that the *TDG* gene was hypermethylated in comparison to normal human plasma cells, resulting in a lower TDG expression and less efficient DNA repair activity in response to hydrogen peroxide-induced DNA damage. The role of TDG in DNA repair was confirmed by compensation of repair capacity after exogenous expression of TDG in the KAS-6/1 MM cell line (106). Other *in vitro* and *in vivo* studies that support the involvement of TDG in cancer showed that TDG regulates the expression of tumor suppressor genes by interacting with several transcription factors, including the retinoic acid receptor (RARa), retinoid X receptor (RXR), estrogen receptor α (ERα), thyroid transcription factor 1 (TTF1), and histone acetyl-transferases p300 and CBP (103, 107–109).

**Figure 3:**
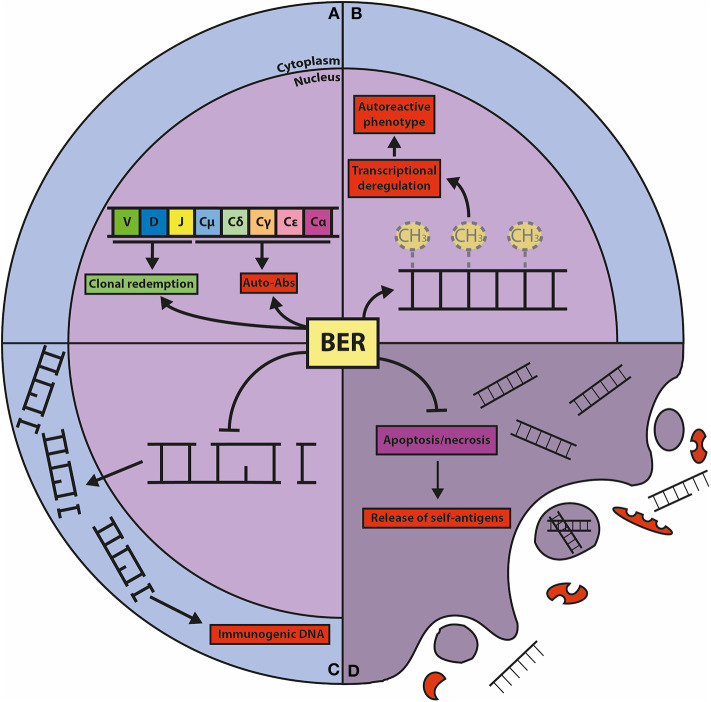
Graphic representation of the known connections between BER and autoimmunity. **(A)** BER is crucially involved in CSR and SHM, processes that shape the effector function and the repertoire of the humoral immune response. CSR is important for the generation of isotype-switched autoreactive antibodies (Auto-Abs), and SHM can result in the clonal redemption of autoreactive B cells by mutating away from self-reactivity. **(B)** The BER glycosylases TDG and MBD4 act as epigenetic regulators of autoimmunity through their DNA demethylating activities. Global and gene-specific DNA hypomethylation is associated with autoreactive features in lymphocytes. **(C)** The repair function of BER guards against damaged DNA in the cytoplasm, which is immunogenic and can elicit (chronic) inflammatory responses and provoke autoreactivity. **(D)** Cells with unrepaired DNA damage dedicated for apoptosis and necrosis release self-antigens that trigger autoimmunity, which is counteracted by the repair and transcriptional functions of BER components.

Dysregulated epigenetic modifications have been implicated in autoimmune diseases. In the *MRL/lpr* mouse model for SLE it was shown that immune cells from lymph node and thymus show lower levels of DNA methylation compared to control mice. In human SLE, DNA hypomethylation was detected in B cells and in autoreactive T cells (110, 111). It was demonstrated that demethylating agents such as 5-azacytidine, procainamide and hydralazine induce autoreactivity in CD4+ T cells from healthy human donors, and provoke SLE-like manifestations in normal mice (111–113). These observations suggest an interesting potential link between BER-mediated DNA demethylation and autoimmune diseases (Figure 3). Mutations in BER genes have indeed been linked to SLE predisposition and lupus-like disease in mice (114–120), however, a direct association between TDG and autoimmune diseases has not been established.

### Methyl-CpG Binding Domain Protein 4 (MBD4)

MBD4 is structurally unrelated to UNG, SMUG1, and TDG, while it shares substrate specificity with these glycosylases. MBD4 can remove T from T:G mismatches at CpG sites, and U, 5hmU and thymine glycol (Tg) when mismatched with G (52) (Table 1; Figure 1). In contrast to *Tdg* knockout mice, *Mbd4* knockout mice are viable and fertile (36), despite their apparent substrate overlap. Overexpression of MBD4 and AID was shown to cause bulk genome demethylation in zebrafish embryos whereas *MBD4* and *AID* knockdown resulted in remethylation of certain genes, suggesting a role for MBD4 in active DNA demethylation, similar to TDG (121). The frequency of C to T transitions at CpG site was increased 3-fold in *Mbd4*^−/−^ mice, indicating that MBD4 is important in preventing mutations at CpG sites (36).

MBD4 expression was induced in mouse splenic B cells activated to undergo CSR. However, targeted deletion of exon 3 or exons 2–5 of *Mbd4* had no effect on CSR or SHM in mice (122, 123). Further analysis indicated that two isoforms of MBD4 are expressed in mouse B cells, a full length and a short form. Strikingly, expression of the short form is retained in the *Mbd4* exon 3 and exon 2–5 deletion-mutant mouse strains, and importantly, glycosylase activity is potentially preserved in this short form (124). This prompted investigators to delete *Mbd4* exons 6–8 in the CH12-F3 mouse cell line that can be induced to undergo CSR, showing that IgA isotype switching was ~4-fold reduced, which was associated with fewer *Ig* S region DSBs. Moreover, sequence analysis of S-S region junctions showed an increase of microhomology-mediated end-joining (MMEJ). The loss of intact MBD4 in these cells resulted in the decreased stability of the MSH2 and MLH1 MMR proteins (122, 124). Corroborating this, MBD4 was identified to interact with MLH1 in a yeast-two-hybrid screen (125). Importantly, splenic B cells from mice that lack MMR proteins show a 2- to 4-fold reduced capacity to undergo CSR due to fewer AID-instigated DSBs (62, 126, 127). Furthermore, splenic B cells from *Mlh1*^−/−^ mice showed increased S-S region microhomology, perhaps because MLH1 diminishes the processivity of EXO1 during patch excision (128). These observations suggest that MBD4 acts in conjunction with MMR during CSR. It was hypothesized that MBD4 is involved in the recognition of U by the MLH1/PMS2 heterodimer and subsequent AP site generation, thereby triggering the recruitment of PCNA, which supports EXO1 recruitment, leading to patch excision and DSB generation (124). The extent to which MBD4 contributes to AP site generation in comparison to UNG has not been studied, but the rather severe reduction in CSR in *Ung*^−/−^ mice suggests that this function of MBD4 is of minor importance. Rather, MBD4 may act as an accessory factor to MMR, facilitating the conversion of distal S region nicks to DSBs. This could be addressed in mice that lack the Sμ tandem repeats (TR), which are more dependent on MMR for CSR, due to loss of closely spaced palindromic AID hotspots (62). We hypothesize that loss of MBD4 has a much greater impact on CSR in these mice. The involvement of MBD4 in SHM was shown by CRISPR/Cas9-mediated deletion of *MBD4* downstream from exon 5 in the chicken DT40 cell line (129). Loss of MBD4 resulted in a significant increase in SHM, mostly focused at C/G bases, but to a much lesser extent than in UNG deficient DT40 cells (130). These results suggest that MBD4 is involved in U removal during SHM and perhaps during CSR, but has a less prominent function than UNG, although it must be noted that these studies were conducted in DT40 cells overexpressing AID, which may have amplified the observed effects. These results await confirmation in a mouse model that lacks expression of the 3′ portion of the *Mbd4* gene.

MBD4 has been implicated in the onset and occurrence of cancer. An increased frequency of intestinal tumors was observed in *Mbd4*^−/−^ *Apc*^*min*/+^ mice, showing increased CpG to TpG mutations in the *Apc* gene (36, 131), although it must be noted that in the Wong et al. (131) study the *Mbd4* exon 3 deletion mutant mouse strain was used, which potentially has retained glycosylase activity (124), raising some doubts on the involvement of MBD4 as a glycosylase in these studies. Nonetheless, MBD4 deficient human tumors carry more SNPs compared to MBD4 proficient tumors (132, 133). DNA repair capacity and cancer incidence were associated with *MBD4* polymorphisms and frameshift mutations (134–137). Of interest, it was suggested that MBD4 exclusively acts as a tumor suppressor in MMR proficient mice as loss of MBD4 did not affect tumor onset or mutation frequency in *Mlh1*^−/−^ or *Msh2*^−/−^ mice (138), although the source of the MBD4 knockout mice in this study, and whether these may have retained glycosylase activity, has not been disclosed. These data suggest a potential epistatic interaction between MBD4 and MMR in cancer, similar to its role in CSR. The involvement of MBD4 in leukemia and lymphoma appears to be rather limited. Loss of MBD4 expression due to germline mutations was detected in a small number of acute myeloid leukemia (AML) patients. These patients displayed a >30-fold increased mutational burden compared to MBD4 proficient AML cases, with the vast majority of mutations being C to T transitions at CpG sites. Furthermore, it was found that hematopoietic stem cell clones with pathogenic mutations in the methyltransferase-coding gene *DNMT3A* repeatedly expanded in the course of the treatment in MBD4 deficient AML patients, suggesting that the loss of MBD4 drives clonal hematopoiesis (139). A fusion transcript that involves the *MBD4* gene and the *PTPRC* gene was identified in a patient suffering from Sézary syndrome, which is an aggressive T-cell lymphoma of the skin (140). The functional consequences of this fusion transcript, and whether it involves aberrant MBD4 activity, remains unexplored.

There are some indications that MBD4 is involved in the pathogenesis of autoimmune diseases by provoking DNA demethylation of costimulatory genes that may lead to aberrant immune activation. In this scenario, MBD4 acts to remove T from T:G mismatches that arise by the deamination of 5mC, effectively erasing this methyl mark (52). In support, it was shown that to DNA hypomethylation in (autoreactive) lymphocyte populations is related to autoimmune diseases (141–143). Moreover, the expression of *MBD4* mRNA showed an inverse correlation with DNA methylation in CD4+ T cells from SLE patients (144), and a positive correlation with the overexpression of costimulatory genes such as *CD40LG, TNFSF7, ITGAL, PRF1*, and *KIR2DL4* (145). It was found that disease progression in MS was associated with elevated *MBD4* gene expression in peripheral blood mononuclear cells (PBMNCs) (146), and an arthritis-linked genomic region that harbors the *Mdb4* gene was identified as a quantitative trait locus in murine collagen-induced arthritis (147). However, a direct role for MBD4 in B cells of (autoantibody-mediated) autoimmune diseases has not been reported.

### Bifunctional Oxidation Damage-Specific Glycosylases

In addition to base removal, bifunctional glycosylases also perform AP lyase activity, cleaving the DNA phosphodiester backbone 3′ to the AP site. As yet, five bifunctional glycosylases have been identified in mammals (OGG1, NTH1, NEIL1, NEIL2, NEIL3), which repair DNA bases damaged by oxidation (148) (Table 1; Figure 1). The AP lyase activity from these glycosylases differs from the AP endonucleases; OGG1, NTH1, and NEIL3 perform a β-elimination reaction on the AP site yielding a 3′ unsaturated aldehyde, whereas NEIL1 and NEIL2 perform an additional δ-elimination reaction converting the 3′ aldehyde to a 3′ phosphate. In contrast, AP endonucleases typically leave a 3′ hydroxyl and a 5′ deoxyribose phosphate (dRP) moiety upon AP site incision (149). These differences in DNA strand incision are important determinants for the ensuing DNA repair. The primary function of OGG1 is to remove 8-oxo-guanine (8-oxoG), an abundant mutagenic base damage caused by the reactive oxygen species (ROS) attack on guanine (150). *Ogg1*^−/−^ mice are viable and fertile but accumulate 8-oxoG and show a moderate (2- to 3-fold) increase in mutations in liver cells (37). Escape from OGG1 excision results in the mispairing of the 8-oxoG lesion with A after replication. The adenine glycosylase MUTYH is responsible for a backup mechanism that excises the mispaired A, and subsequent repair of the AP site and incorporation of C provides another opportunity for OGG1 to excise the 8-oxoG, thereby repressing C to T and G to A transitions (32). *Mutyh*^−/−^ mice are born at the expected Mendelian ratio and develop normally but are susceptible to oxidative stress and predisposed to develop tumors (38, 151). NTH1 is involved in the repair of thymine glycol (Tg), generated by ROS-mediated oxidation of thymine (39, 152). Homozygous *Nth1* mutant mice show no gross developmental abnormalities but displayed a slower Tg turnover in the liver (39). Oxidized pyrimidines (C, T, U) are recognized and repaired by NEIL1 and NEIL2, but the preferred substrate for NEIL1 and NEIL2 are hydantoin lesions, which are derived from progressively oxidized 8-oxoG (153). NEIL1 and NEIL2 favor ssDNA structures such as bubbles and loops. NEIL1 associates with replication forks and is involved in pre-replicative repair in S-phase (154), whereas NEIL2 is mostly involved in repair at transcribed genes (155). Both NEIL1 and NEIL2 are involved in the maintenance of mitochondrial (mt) DNA, similar to OGG1 and NTH1 (156). *Neil1*^−/−^ mice are born at the expected Mendelian rates and show no developmental defects. However, these mice show an accumulation of mtDNA damage and develop a severe metabolic syndrome by 6–10 months of age, characterized by obesity, fatty liver disease, and kidney vacuolization. It was hypothesized that mtDNA damage hampers replication and transcription of mitochondrial genes involved in metabolism, thereby impairing energy homeostasis (40). *Neil2*^−/−^ mice are viable and show no overt phenotype, they accumulate oxidative DNA damage preferentially in transcribed regions, as expected. *Neil2*^−/−^ mice display an increased responsiveness to inflammatory stimuli such as intranasal challenges with LPS, glucose oxidase (GOx), or tumor necrosis factor α (TNFα) (41). How these glycosylases are involved in innate inflammatory responses remains to be characterized. NEIL3 has an extended c-terminal tail compared to NEIL1 and NEIL2 and its properties were only quite recently characterized, showing that it recognizes hydantoins and 2,6-diamino-4-hydroxy-5-formamidopyrimidine (FapyG) in ssDNA in bubble structures, similar to NEIL1 and NEIL2, but uses a different AP lyase reaction mechanism (157). NEIL3 was found to be expressed predominantly in hematopoietic and lymphoid tissue, and in the brain (42, 158, 159). *Neil3*^−/−^ mice are viable and fertile (42).

Typically, the bifunctional glycosylases have no major role in the AgR diversification mechanisms. OGG1 and NTH1 were demonstrated not to contribute to CSR (160). The role of MUTYH in CSR and SHM has not been investigated. *Ogg1*^−/−^ mice displayed normal SHM, and OGG1 was shown not to be upregulated in mouse GC B cells (161), whereas it was increased in human GC B cells that undergo SHM (162). This discrepancy may be related to the chronic nature of GCs in human tonsil, accompanied by continuous apoptosis and release of oxidation products, while induced GCs in mouse tissues are transient. In contrast, *Neil1*^−/−^ mice showed moderate (<2-fold) decrease in GC B-cell expansion after immunization with the model antigen nitrophenylactetyl-chicken γ-globulin (NP-CGG). Accordingly, the mutation frequency in the JH4 intron was slightly decreased (~80% of wildtype) in *Neil1*^−/−^ mice (163). These results suggest that NEIL1 is required to curb endogenous oxidative damage related to the rapid expansion of GC B cells, but does not participate in the repair phase of AID-instigated DNA lesions. In agreement, *NEIL1* was identified as a likely candidate gene for common variable immunodeficiency in a patient with 15q24 deletion (164). *Neil3*^−/−^ mice showed a similar modest decrease in GC B-cell expansion (<2-fold), due to apoptosis. However, SHM was not significantly affected in these mice (116). Interestingly, 3 siblings from a consanguineous family were identified that carried a homozygous missense mutation in the *NEIL3* gene, suffering from fatal infections and impaired B-cell function. Studies conducted on B cells from one of these patients showed a decreased capacity to undergo IgG and IgE CSR *in vitro* (116). These results underscore that the bifunctional glycosylases do not directly participate in the AgR diversification mechanisms, but are essential for the fitness of cells that undergo these processes.

Most of the bifunctional glycosylases are linked to cancer. Human tumor tissues were shown to accumulate clustered DNA lesions due to oxidative damage, suggesting impaired function of oxidative repair glycosylases and/or increased generation of oxidative agents (165). Moreover, knockout mouse models showed increased tumorigenesis with a differential tumor spectrum, likely reflecting tissue-specific dependencies. Aging *Ogg1*^−/−^ mice accumulate genomic 8-oxoG and develop lung cancer (166), whereas most *Mutyh*^−/−^ mice spontaneously develop intestinal tumors, which was increased by treatment with an oxidizing agent (151). In agreement, reduced activity and functional polymorphisms of *OGG1* are associated with various types of human cancer, including lung cancer (167–169), and mutations in the *MUTYH* gene confer a heritable form of colorectal cancer predisposition (170). Of interest, in comparison to *Ogg1*^−/−^ mice, *Ogg1*^−/−^ *Mutyh*^−/−^ mice were further predisposed to develop cancer, presenting with predominantly lung and ovarian tumors, and B-cell lymphomas (38). In contrast, B-cell lymphomas that arise in a *Msh2*^−/−^ mouse strain depended on MUTYH, as lymphomagenesis was significantly delayed in *Msh2*^−/−^ *Mutyh*^−/−^ mice (171). These results underscore differential interdependencies of these BER components acting in a context-specific manner. Reduced expression of *NTH1* mRNA was found in 36% of primary gastric carcinomas (172). It was shown that a functional germline variant of *NTH1* that is expressed in a sizeable fraction of the human population (6%) causes genomic instability and cellular transformation in an experimental setting (173). It was reported that the expression of NEIL1 and NEIL2 inversely correlated with the number of somatic mutations in several cancers, whereas NEIL3 showed a positive correlation (174). Ectopic expression of a rare human *NEIL1* germline variant devoid of glycosylase activity induced replication stress, DNA breaks and anchorage-independent growth, suggesting that it confers an increased risk for cancer (175). However, the (combined) deficiencies for the NEIL glycosylases (double and triple knockout mice) did not result in increased mutations or cancer predisposition in mice under normal conditions (176). These results suggest that there is limited overlap in the functions of the NEIL glycosylases. It remains to be addressed whether exposure to oxidative agents induces tumor formation in these mice. Several sporadic reports suggest the involvement of oxidative damage glycosylases in B-cell lymphomas. For instance, a deletion of the *NEIL1* gene was found in a case of FL that transformed into a B-cell acute lymphoblastic leukemia (B-ALL) (177), and the expression of *NEIL1* mRNA was part of a classifier that distinguished the germinal center B-cell-like (GCB) subtype from the activated B-cell-like (ABC) subgroup of DLBCL, and was associated with disease aggressiveness in the ABC subgroup (178). However, a clear role in B-cell lymphomas has not been reported, besides as a backup downstream of MMR defects (171). Most B-cell lymphomas are driven by genomic aberrations and mutations that are related to the GC response and *Ig* diversification mechanisms (179), the fact that the bifunctional glycosylases are not directly involved in *Ig* diversifications might explain the limited role of these enzymes in lymphomagenesis.

Oxidative DNA damage can provoke an inflammatory reaction by activation of innate immune receptors, which may ultimately trigger an immune response to self-antigens (180) (Figure 3). The glycosylases involved in mtDNA repair are especially relevant in this process, as these counteract the oxidation and release of mtDNA that activates the inflammasome, resulting in release of proinflammatory cytokines and mediators (181). In agreement, there is some evidence that SLE patients have increased levels of DNA damage compared to normal individuals, indicating that ROS-induced DNA damage and decreased OGG1 expression are involved in the development of SLE (182–184). In addition, there are several reports showing that *OGG1* polymorphisms are associated with autoimmune diseases such as SLE, RA, and MS (115, 185–187). Also, two *MUTYH* polymorphisms were found to be associated with RA (188). *Neil2*^−/−^ mice show no signs of autoimmune diseases, but develop severe lung inflammation upon intranasal challenge with LPS, which is associated with an accumulation of DNA damage and apoptosis that triggers local inflammation in the lung (41). The association with autoimmune disease appears especially strong in the case of NEIL3. *Neil3*^−/−^ mice display elevated levels of autoantibody levels and develop nephritis when challenged with the immunostimulant poly(I:C). It was hypothesized that increased apoptosis of GC B cells from Peyer's patches and splenic B and T cells observed in *Neil3*^−/−^ mice could stimulate autoreactivity due to release of self-antigens (Figure 3). Concordantly, NEIL3 deficient patients developed severe autoimmunity and suffered from fatal recurrent infections (116). These results indicate that NEIL3 is of crucial importance for the protection against autoimmunity. Importantly, the homozygous missense mutation found in the *NEIL3* gene (D132V) in the consanguineous NEIL3 deficient patients is present in about 2% of healthy individuals from Middle Eastern descent (116), but whether this confers an increased risk to develop autoimmune disease remains to be studied.

## AP Endonucleases

The AP endonucleases act after base removal by DNA glycosylases by nicking the DNA phosphodiester backbone. The AP endonucleases are grouped into two major groups according to their modes of action (Table 1; Figure 1). The abovementioned bifunctional glycosylases belong to class I, whereas the predominant AP endonucleases in most organisms belong to class II, which are AP endonucleases that cleave the DNA by a hydrolytic mechanism leaving a 3′ hydroxyl and a 5′ dRP group (189). In most cases, class I AP lyase requires the subsequent activity of class II AP endonucleases to remove 3′ blocking moieties and allow DNA polymerase-mediated repair synthesis (190). These non-redundant activities of class II AP endonucleases are of vital importance for cellular growth, preventing apoptosis by restoring (endogenous) DNA damage (191).

### AP Endonuclease 1 (APE1)

In humans, two AP endonucleases are identified, APE1 and APE2, of which APE1 is predominantly active, accounting for >90% of AP site repair (192). APE1 is ubiquitously expressed, showing nuclear protein expression in all tissues. It appears that APE1 has a dual function, being involved in BER and acting as a transcriptional regulator by serving as a reducing donor for oxidized cysteines that hamper DNA binding of several transcription factors (193). *Ape1* gene targeting in the mouse resulted in embryonic lethality, showing that APE1 is required for early embryonic development (43, 194). However, heterozygous *Ape1*^+/−^ mice are viable but display haploinsufficiency, showing sensitivity to oxidative stress and increased mutations (195, 196). B-cell development is unaltered in *Ape1*^+/−^ mice, and APE1 is expressed in resting and activated B cells. Of interest, *ex vivo* CSR was found to be modestly reduced (<2-fold) in *Ape1*^+/−^ B cells compared to B cells from wildtype littermates. Moreover, a small molecule inhibitor of APE1 reduced CSR *in vitro*, independently of its effect on cell proliferation (44, 197). Consistent with the study from Guikema *et al.*, further reduction to one copy of *Ape1* in the CH12-F3 cell line, which normally expresses 3 copies, reduced the APE1 protein level by 60% and reduced IgA CSR by 50%. Importantly, deletion of all 3 copies of *Ape1* reduced CSR efficiency to 20% of the wildtype level providing direct evidence that APE1 is required for efficient CSR (198). Fewer Sμ DSBs were observed in *Ape1*^+/−^ B cells undergoing CSR, strongly suggesting that APE1 is responsible for the incisions at AP sites that are generated by AID and UNG during CSR (44). Interestingly, using a specific small molecule inhibitor it was suggested that the redox function of APE1 also plays a role in CSR by regulating interleukin-6 signaling and IgA expression in the CH12-F3 cell line (199). Recently, the dogma that CSR takes place within the GC was challenged by use of an elegant adoptive transfer mouse model. It was shown that CSR mostly occurs outside of the GC, prior to the onset of SHM (200). Having shown that CSR depends on AID, UNG and APE1 to generate S regions DSBs (4, 33, 44), it was demonstrated that APE1 expression is downregulated in GC B cells, thereby preventing CSR in the GC (200, 201). In agreement, APE1 does not seem to be required for SHM, which takes place in the GC (198, 201–203) (Figure 2).

The expression of APE1 and its subcellular localization are associated with various types of cancer and the response to therapy (204–209). Several polymorphic *APE1* variants that show reduced DNA incision activity are linked to cancer risk and therapy sensitivity (210–213). There is no clear indication that APE1 is implicated in lymphomagenesis. However, the established relation between B-cell lymphomas and CSR-mediated chromosomal translocations (214), and the function of APE1 therein, suggest that APE1 functions as a crucial mediator of DSBs downstream of AID, which when repaired illegitimately lead to lymphoma-associated genomic lesions. In addition, the redox function of APE1 was shown to be a therapeutically amenable disease determinant in T-cell acute lymphoblastic leukemia (T-ALL) and AML (215–217).

A few reports indicate that APE1 may be involved in autoimmunity. The high mobility group box 1 protein (HMGB1) is released from necrotic cells, which triggers an inflammatory response in effector cells. Interestingly, the cytoplasmic expression of APE1 dampened this inflammatory response in monocytic cells, most likely mediated by its transcriptional regulatory function (218). In addition, APE1 is one of the nuclear target proteins for autoantibodies in SLE (219). It is conceivable that the role of APE1 in autoimmune disease is mainly related to its function in preventing DNA damage that may drive aberrant immune responses (220) (Figure 3).

### AP Endonuclease 2 (APE2)

APE2 is a nuclear protein closely homologous to APE1 but with a weaker endonuclease activity (221). However, APE2 possesses strong 3′-5′ exonuclease and 3′-phosphodiesterase activity stimulated by the interaction with PCNA (222, 223). APE2 is involved in the repair of oxidative DNA damage by acting on damaged and mismatches DNA 3′ ends, removing A opposite of 8-oxoG (223). Additional evidence that APE2 participates in the response to oxidative DNA damage was provided, showing that the 3′-phosphodiesterase and 3′-5′ exonuclease activities are required for end resection, thereby stimulating homologous recombination-mediated repair (HR). Additionally, it was shown that APE2 participates in the ATR-CHK1 cell cycle checkpoint, facilitating CHK1 phosphorylation in response to oxidative DNA damage (224). Circumstantial evidence suggests that APE2 might also be involved in NHEJ, perhaps by serving as an end-cleaning/processing enzyme by virtue of its 3′-phosphodiesterase activity (225). Interestingly, the endonuclease activity of APE2 was shown to be crucial for the survival of cells with defective HR due to BRCA2 deficiency, whereas APE1 was not required (226).

APE2 deficient mice develop normally but show a 2-fold decrease in pre-B cell production in the bone marrow (227), and a similar reduction in newly formed and follicular B-cells in the spleen, but APE2 was shown not to be directly involved in V(D)J recombination. In addition, the expansion of early B-cell progenitors during the recovery after a chemotherapeutic challenge was hampered in APE2 deficient mice. Interestingly, B-cell cellularity was not altered in *Ape2*^*Y*/−^ *tp53*^−/−^ mice indicating that the loss of B cells was due to p53-dependent cell death triggered by DNA damage (228). It was found that APE2-deficient activated B cells are hypersensitive to oxidative damage, indicating that APE2 protects proliferating B cells from intracellular ROS (228). The expression of APE2 is upregulated in GC B cells, and APE2 deficient mice have ~2.5-fold fewer GC B cells due to the accumulation of AID-independent DNA damage. However, B-cell selection in the GC and affinity appeared not to be affected in APE2-deficient mice. The reduction in GC B-cell frequency was found to be related to AID-independent DNA damage resulting in the reduced expression of BCL6, which is required for the development of GC B cells (228). APE2 was shown to contribute to CSR in splenic B cells, participating in the formation of S region DSBs (44), however, its contribution seems to be of minor importance compared to APE1, as APE1 is sufficient for CSR, especially in cell lines that can be induced to undergo CSR (198, 202). However, in contrast to APE1, APE2 clearly participates in the SHM process, since APE2 deficient mice have a 2-fold reduced mutation frequency and altered mutation spectrum, with a significant reduction in mutations at A:T base pairs (59% of JH4 intron mutations wildtype mice versus 48% in APE2 deficient mice). It was suggested that APE2 contributes to DNA nicks that provide entry points for EXO1 that instigates ncMMR-mediated A:T mutations (201, 202). In addition, the 3′-5′ exonuclease activity of APE2 may also support SHM by the excision of short DNA patches that are targeted by AID and/or facilitate ncMMR (229) (Figure 2).

A recent study reported on the dysregulation of APE2 in a multitude of human cancers, showing that ~17% of cancer cases have genomic alterations involving APE2, mostly consisting of heterozygous deletions and gains. Liver, skin, and breast cancer showed the highest frequency (~24%), and somatic mutations in APE2 were found in uterine, skin, and lung cancers. Many of these mutations were speculated to affect PCNA and ssDNA binding, thereby reducing exonuclease activity. Moreover, *APE2* mRNA levels were found to be upregulated in several cancer subtypes and showed a positive correlation with DNA damage response (DDR) genes (230). These data suggest that APE2 might be involved in cancer development, although direct functional evidence is not provided. These observations are in line with the notion that BER activity in tumors determines prognosis and response to therapy (231). A recent study reported on the upregulation of APE1 and APE2 expression in MM. Interestingly, knockdown of APE2 compromised HR proficiency of MM cells, consistent with the previously reported role of APE2 in HR (224, 229, 232).

Indications that APE2 is directly involved in other B-cell neoplasms or in autoimmune diseases have not been reported, but it's evident association with CSR and SHM indicate that APE2 has a role in genomic alterations in B-cell lymphomas and aberrant clonal selection in autoantibody-mediated diseases.

## SINGLE Strand Nick Processing BER Enzymes

AP endonuclease- and AP lyase-generated nicks are processed by non-displacement DNA synthesis (short-patch BER) or displacement synthesis (long-patch BER). The mechanistic basis for the choice between these pathways involves various factors but is still poorly understood (233). Short-patch BER involves one nucleotide insertion by DNA polymerase β (POLB), whereas long-patch BER can be 2–16 nucleotides, executed by POLB or DNA polymerase δ/ε (POLD/E), stimulated by PCNA (234). The latter involves the trimming of overhanging ends by flap endonuclease 1 (FEN1) (Table 1; Figure 1).

### DNA Polymerase β (POLB)

POLB has a dual catalytic function and is uniquely involved in BER. The C-terminal part of POLB is involved in DNA synthesis, while an independently folded N-terminal region is required for dRP lyase activity (235). POLB is the major lyase in the repair of oxidized and alkylated bases (236, 237). Although POLB lacks intrinsic 3′-5′ proofreading activity, it has a relatively low error rate in both short-patch as long-patch BER (238). Moreover, the ensuing DNA ligation step is inefficient when POLB inserts a mismatched or damaged base, providing an additional safety measure to prevent BER associated mutagenesis (239). *POLB* is an essential gene, mice with a heterozygous germline *Polb* deletion do not generate *Polb*^−/−^ offspring. Embryos homozygous for the *Polb* deletion die at days 18–19 post-coitum (45). The structurally related DNA polymerase λ (POLL) contributes to BER as a backup for POLB but is apparently not sufficient for development (240). However, POLB deficient cell lines can be obtained by gene targeting and have indicated that POLB is required for BER (241).

Cell type-specific gene targeting by the Cre/Lox system and adoptive transfer of *Polb*^−/−^ fetal liver cells have enabled the characterization of the role of POLB in lymphoid cells (45, 242). Using these approaches, it was shown that POLB is not required for T-cell or B-cell development, suggesting that POLB is not critically involved in V(D)J recombination (45, 242). It was shown that POLB is able to repair AID-instigated DNA nicks in S regions, thereby inhibiting CSR. However, this was only apparent for IgG2a, and to a lesser extent for IgG2b and IgG3 isotype switching. Of interest, the S regions located upstream of these specific constant regions contain the lowest density of AID hotspot motifs (especially for IgG2a), and it was hypothesized that AID-induced nicks may be limiting in these S regions. These results suggest that BER activity is overwhelmed by AID, and that POLB fails to repair all S region lesions during CSR (243). Furthermore, it was demonstrated that the GC response is normal in mice reconstituted with POLB deficient cells, showing unaltered mutation spectrum and levels, indicating that POLB is not involved in SHM, as was also the case for POLL (242, 244). When induced to undergo IgA CSR, the CH12-F3 cell lines accrue AID-dependent mutations in Sμ. However, the frequency of mutations at A:T base pairs is much lower in this cell line, as it true for other *in vitro* systems used to study AID-instigated mutagenesis, compared to *in vivo* B cells (0–20 vs. 50–60% of all mutations, respectively) (245–248). We have recently shown that knockdown of POLB in CH12-F3 cells, and in fibroblasts engineered to express AID, resulted in a 3-fold increase in A:T mutagenesis, which was associated with increased recruitment of MMR components (18) (preprint). Moreover, we have demonstrated that GC B cells *in vivo* lack POLB protein expression, in contrast to *in vitro* cultured cells, which functionally express POLB. This in part explains the lack of a GC phenotype in mice with POLB deficient lymphocytes. Furthermore, our studies suggest that POLB protein is destabilized in GC B cells due to the hypoxic microenvironment that marks the GC (18, 249, 250). Our data suggests that the specific loss of POLB in GC B cells is instrumental in the mutagenic repair of AID-dependent lesions (Figure 2). We speculate that the overall loss of POLB in combination with dUTP misincorporation related to DNA replication, and spontaneous base deaminations destabilize the genome in GC B cells, thereby potentially driving the development of GC B-cell derived lymphomas.

In line with this, the Sweasy group has identified and characterized various functional POLB variants that have been implicated in the development of cancer (251–255). It was reported that POLB variants can be detected in up to 30% of human cancers (256). These variants either have reduced repair activity or decreased fidelity. Importantly, expression of several of these variants resulted in cellular transformation, suggesting that these mutations are sufficient to drive cancer development (251). Moreover, *Polb* haploinsufficiency increased cancer incidence in aging mice. Of interest, ~40% of aging *Polb*^+/−^ mice showed lymphoid hyperplasia, and aged *Polb*^+/−^ mice had a 7-fold increase in lymphoma incidence compared to wildtype mice (257). These tumors develop without an obvious increase in mutation frequency, which would suggest that DSBs accumulate in these mice. For human B-cell neoplasia, a functional *POLB* P242R non-synonymous SNP, which bestows a slower catalytic activity, was identified as an independent prognostic marker in B-CLL patients (258, 259).

There is a potential link between POLB and autoimmune diseases. A genome-wide association study has identified an association between SLE and a functional *POLB* SNP that results in lowered POLB expression (260). In agreement, a mouse model with the hypomorphic Y265C *POLB* allele developed a lupus-like syndrome characterized by anti-nuclear antibodies, glomerular nephritis, and cervical lymphadenopathy (117). BCR repertoire analysis showed that the hypervariable complementarity determining region 3 (CDR3) is significantly shorter in bone marrow progenitor B cells and mature splenic B cells from *POLB* hypomorphic mice. No alterations in CSR were found in *ex vivo* activated splenic B cells from these mice. In addition, *POLB* hypomorphic mice displayed a significant increase in SHM frequency with increased transversions at G:C base pairs and increased A:T mutagenesis. The number of GCs were also increased in these mice. It was suggested that POLB participates in the V(D)J recombination by processing DNA ends and preventing nuclease activity prior to joining, thereby affecting CDR3 length. This role may have been overlooked in the adoptive transfer experiments that suggested POLB is not involved in V(D)J recombination (242). However, the relevance of this phenotype for the development of lupus remains unexplained. The effects of the hypomorphic *POLB* allele on SHM are difficult to reconcile with our findings showing that POLB is limiting in GC B cells due to low protein stability (18) (preprint). It might be that the hypomorphic mutant POLB has an increased stability in GC B cells, despite hampered catalytic activity. Perhaps mutagenic translesion (TLS) repair is favored in GC B cells expressing the mutant (stabile) POLB, as it is possible that the POLB mutant is recruited to AID-instigated nicks, but is not processive, thereby provoking the recruitment of TLS polymerases. Finally, it was hypothesized that the increased turnover and apoptosis in GCs in hypomorphic *POLB* mice increases the exposure to self-antigen, causing autoimmune disease (Figure 3).

### Flap Endonuclease 1 (FEN1)

FEN1 is required for the removal of the displaced DNA strand in long-patch BER, and the processing of Okazaki fragments during lagging strand DNA synthesis (261). In addition, FEN1 is of crucial importance for MMEJ by cleaving the displaced 5′-flaps prior to joining (262), and can act as a 5′-3′ exonuclease to trim DNA ends during HR (263) (Table 1; Figure 1). Mice deficient for FEN1 could not obtained due to embryonic lethality, however, *Fen1*^+/−^ mice develop relatively normal. *Fen1*^+/−^ mice show diminished lymphocyte cellularity and premature thymic involution, likely related to the essential role of FEN1 in DNA synthesis and replication (46).

Interestingly, *Fen1* haploinsufficiency accelerated cancer progression in *Apc* mutant mice, leading to an increased number of intestinal adenocarcinomas, characterized by microsatellite instability (MSI) (46). Knock-in of a cancer-related inactivating *Fen1* mutation resulted in a high incidence of lung carcinomas (120). Overall, FEN1 overexpression as well as loss of function have been associated with human cancers, likely related to the dual nature of FEN1, being involved in DNA replication and in DNA repair (264). Moreover, functional defects in FEN1 were linked to increased risk in human cancer types (265–267). A considerable proportion (17%) of *Fen1*^+/−^ mice developed B-cell lymphomas. In agreement, female mice with a compound *Fen1* mutation in the nuclease domain developed B-cell lymphomas originating from GC B cells. The exact reason for this gender bias in lymphomagenesis is not understood, but might be related to the immunomodulatory effects of estrogen (268). B cells from these mice show normal *ex vivo* CSR and *in vivo* SHM, suggesting that FEN1 is not directly involved in *Ig* diversification-driven chromosomal aberrations linked to B-cell lymphomas, but rather, is involved in preventing DNA damage that is associated with the rapid proliferation of B cells in the GC.

A nuclease-deficient *Fen1* mutant mouse model displayed a marked predisposition to chronic inflammation and autoimmunity (120). It was shown that the *Fen1* E120D mutant mice accumulate undigested DNA in apoptotic cells, which may be at the basis of uncontrolled inflammatory responses by activation of stimulator of interferon genes (STING) signaling, which is linked to the development of autoimmune diseases (269, 270) (Figure 3). In agreement, *Fen1* E120D mutant mice showed increased levels of anti-nuclear and anti-dsDNA antibodies (120). Moreover, the E120D mutation was found more frequently in lupus nephritis patients compared to healthy individuals (271).

### DNA Ligase I and III

After nick processing, the DNA is sealed by DNA ligase, thereby completing BER. DNA ligase III (LIG3) is responsible for the nick-sealing step in short-patch BER (272), whereas DNA ligase I (LIG1) ligates the nick in long-patch BER (273) (Table 1; Figure 1). The accessory factor X-ray cross-complementing protein 1 (XRCC1) interacts with LIG3, serving as a scaffold protein that organizes repair by binding POLB. LIG1 plays an essential role in DNA replication by joining Okazaki fragments to extended DNA during lagging strand DNA synthesis (274). Both LIG1 and LIG3 were shown to be involved in MMEJ (275), whereas LIG3 and is the only DNA ligase that functions in mitochondria, where it acts to maintain mtDNA integrity independently of XRCC1 (276, 277). LIG1 and LIG3 are essential for embryonic development (47, 48). However, cell lines lacking LIG1 could be obtained due to the functional redundancy with LIG3 and LIG4, of which the latter mainly functions in NHEJ (278–280). Interestingly, biallelic *LIG1* mutations were identified in 5 human subjects, these patients suffered from a spectrum of immune deficiencies characterized by lymphopenia, likely resulting from defective DNA repair in B cells and T cells (281).

Gene targeting in the CH12-F3 cell line has demonstrated that LIG1 and LIG3 are not required and act redundantly in CSR (278, 282), whereas loss of LIG4 decreased CSR, likely due to its central role in NHEJ (282). Interestingly, using an indirect approach it was shown that LIG1 is the major contributor to MMEJ-mediated CSR to IgG1, conditional deletion of *Xrcc1* and *Xrcc4* in mature B cells had no significant impact on CSR, whereas LIG3 expression was severely reduced in *Xrcc1/4* deficient B cells (283). *LIG1* mRNA is highly expressed in human tonsillar GC B cells compared to naïve B cells, whereas *LIG3* mRNA expression was not found to be increased in GC B cells (284). The roles of LIG1 and LIG3 in SHM have not been directly assessed. However, a *LIG1* deficient patient showed a much lower level of *IGH* somatic mutations in peripheral blood mononuclear cells, perhaps caused by blunted GC responses (281). Based on our findings we propose that AID/UNG/APE1-generated nicks are shunted toward error-prone repair by the ncMMR system, as POLB is limiting in GC B cells, thereby bypassing (short-patch) BER and nick-sealing by LIG3. LIG1 is responsible for sealing the nick in MMR-mediated patch repair and would be indirectly involved in SHM in this scenario (285). Of interest, SHM was increased in Peyer's patch GC B cells from *Xrcc1*^+/−^ mice, which develop normally but are haploinsufficient. No obvious alteration in the SHM spectrum was observed in these mice (286). These data suggest that BER has limited access to AID-instigated lesions, causing uracils to escape faithful repair and become substrates for mutagenic repair.

Overexpression of each of the DNA ligases has been found in cancers, which is typically related to increased proliferation and reliance on DNA end-joining due to (therapy-induced) DNA damage (287). LIG3 was shown to promote chromosomal translocations in cell lines upon induction of distal DSBs (288). Increased expression of LIG1 was linked to genomic instability by causing trinucleotide repeat instability due to reduced slipped DNA repair (289). On the other hand, loss of LIG1 function has also been associated with cancer predisposition (290). Moreover, DNA ligases are under consideration as targets for anticancer therapy (291, 292). LIG1 and LIG3 have not been directly associated with lymphomagenesis, but it was demonstrated that C-MYC, which is frequently deregulated in B-cell lymphomas and MM, drives the expression LIG3 (293, 294). Given their role in MMEJ, which is responsible for illegitimate recombinations, LIG1 and LIG3 are likely to be of pivotal importance for genomic instability and clonal evolution in B-cell malignancies. In agreement, it was shown that MMEJ repair activity was increased in B-CLL cells compared to normal B cells (295).

The involvement of LIG1 and LIG3 in autoimmune diseases has not been reported, however, it is conceivable that LIG1 and LIG3 may prevent autoimmunity as they are crucial for the maintenance of mitochondrial and nuclear DNA, which can have immunostimulatory effects that may underlie a breach of clonal tolerance (Figure 3).

## Concluding Remarks

In lymphocytes, strategies have evolved that both utilize the faithful nature of BER to ensure genetic integrity, as well as subvert it to allow (localized) mutagenic repair for AgR diversifications. Many of the key players of the ubiquitous BER pathway are of essential importance for AgR diversification mechanisms that shape the adaptive immune response, and are at the basis of B-cell neoplasia and autoimmune diseases. In that sense, the BER pathway cannot be regarded in isolation, as it acts in concert with other repair pathways. For instance, the specific loss of the late gap-filling step of short-patch BER in GC B cells favors mutagenic repair by ncMMR, thereby contributing to diversification. However, mutagenic repair is also responsible for oncogenic mutations and rearrangements that drive the development and progression of B-cell malignancies. BER can thus be regarded as a double-edged sword that on the one hand protects the organism by ensuring highly specific adaptive immune responses and on the other hand jeopardizes it by provoking genomic instability. In that regard, the BER pathway is also of pivotal importance to minimize the release of immunogenic DNA that can instigate autoimmune diseases. Here too, the double-edged nature of the BER pathway is apparent (Figure 2). BER is instrumental in the development of autoantibodies, but at the same time prevents the release of (extracellular) DNA and DNA-protein complexes that (hyper) activate cytosolic and endosomal DNA sensors leading to auto-inflammatory and autoimmune diseases (Figure 3). From that point of view, the development of B-cell neoplasia and autoantibody-driven autoimmunity can have a common etiology as in both cases it relies on the evasion of checkpoints that either safeguards genomic integrity or prevents autoimmunity. Recent experimental and clinical data has suggested that these checkpoints may overlap, since patients with autoimmune diseases have an increased risk to develop B-cell lymphomas (296–298), and several B-cell lymphoma subsets are characterized by the expression of autoreactive BCRs (299–303). In addition, lymphoma driver mutations have been detected in rare B cells that produce pathogenic autoantibodies from primary Sjögren's syndrome patients, underscoring a potential common origin for B-cell lymphoma and autoimmune disease (304). BER has been linked to these conditions, acting to both prevent and to provoke disease, depending on the context and timing. These mechanisms will remain the subject of intense research in the coming years, which will provide important new insight into the complexity of biological systems that drive adaptive immunity and related diseases.

## Author Contributions

MS, TD, and JG wrote and edited the manuscript and generated figures.

## Conflict of Interest

The authors declare that the research was conducted in the absence of any commercial or financial relationships that could be construed as a potential conflict of interest.
